# Injectable Enzymatically Hardened Calcium Phosphate Biocement

**DOI:** 10.3390/jfb11040074

**Published:** 2020-10-12

**Authors:** Lubomir Medvecky, Radoslava Štulajterová, Maria Giretova, Lenka Luptakova, Tibor Sopčák

**Affiliations:** 1 Institute of Materials Research of SAS, Watsonova 47, 04001 Kosice, Slovakia; rstulajterova@saske.sk (R.Š.); mgiretova@saske.sk (M.G.); tsopcak@saske.sk (T.S.); 2 Institute of Biology, Zoology and Radiology, University of Veterinary Medicine and Pharmacy in Kosice, Komenskeho 73, 04181 Kosice, Slovakia; lenka.luptakova@uvlf.sk

**Keywords:** calcium phosphate cement, phytic acid, polyacrylic acid, setting process, in vitro

## Abstract

(1) Background: The preparation and characterization of novel fully injectable enzymatically hardened tetracalcium phosphate/monetite cements (CXI cements) using phytic acid/phytase (PHYT/F3P) hardening liquid with a small addition of polyacrylic acid/carboxymethyl cellulose anionic polyelectrolyte (PAA/CMC) and enhanced bioactivity. (2) Methods: Composite cements were prepared by mixing of calcium phosphate powder mixture with hardening liquid containing anionic polyelectrolyte. Phase and microstructural analysis, compressive strength, release of ions and in vitro testing were used for the evaluation of cement properties. (3) Results: The simple possibility to control the setting time of self-setting CXI cements was shown (7–28 min) by the change in P/L ratio or PHYT/F3P reaction time. The wet compressive strength of cements (up to 15 MPa) was close to cancellous bone. The increase in PAA content to 1 wt% caused refinement and change in the morphology of hydroxyapatite particles. Cement pastes had a high resistance to wash-out in a short time after cement mixing. The noncytotoxic character of CX cement extracts was verified. Moreover, PHYT supported the formation of Ca deposits, and the additional synergistic effect of PAA and CMC on enhanced ALP activity was found, along with the strong up-regulation of osteogenic gene expressions for osteopontin, osteocalcin and IGF1 growth factor evaluated by the RT-qPCR analysis in osteogenic αMEM 50% CXI extracts. (4) Conclusions: The fully injectable composite calcium phosphate bicements with anionic polyelectrolyte addition showed good mechanical and physico-chemical properties and enhanced osteogenic bioactivity which is a promising assumption for their application in bone defect regeneration.

## 1. Introduction

Calcium phosphate biocements (CPC) represent biomaterials which are continuously improved in terms of their positive biological properties like biocompatibility, osteoinductivity, osteoconductivity as well as other physico-chemical or mechanical properties. CPCs are characterized by specific properties, which predetermine their perfect filling to various types of bone defects in the maxillofacial, cranial area [1,2], dentistry and orthopedics [3]. For the above applications, a considerable degree of plasticity especially in the stage of cement paste is a very important characteristic. The final products of CPC hardening are the specific forms of nanocrystalline or amorphous calcium deficient hydroxyapatite (HAP), which support the activity of specific bone cells (osteoclasts, osteoblasts) and promote the cell proliferation and growth not only due to a rise in the concentration of calcium and phosphate ions around the implanted biocement, but also the presence of a large proportion of micropores in the microstructure with corresponding large specific surface area. The appropriate microstructure provides suitable conditions for the adsorption of various types of cytokines, proteins (e.g., morphogenic proteins, extracellular adherins) stimulating the biological activity of cells. The applicability of CPC in the bone defect treatment in practice can be significantly improved if cement pastes are injectable and strongly adherent to the surrounding tissue. In practice, three types of injectable cements are used, based on methacrylate composites (presence of free monomer, low bioactivity, non-resorbable, high temperature during polymerization), calcium sulfate cements (rapidly resorbable and increased solubility in body fluids) and calcium phosphate cements (problems with pH, longer setting time, problematic viscoelastic properties) [4]. The highest biocompatibility (not only from the aspect of low irritation of the surrounding tissues, but also chemical, phase and structural compatibility, low cytotoxicity) was achieved in the case of CPC. Previously used injectable CPC pastes were not only composed of the standard calcium phosphates such as tetracalcium phosphate, tricalcium phosphate, brushite with additions of HAP and calcium carbonate but also contained phosphate solution which can contain polymerizable components, e.g., bisGMA resp. methacrylate. The effect of chitosan, starch, gelatin or silanization as well as the addition of bioactive glasses on the mechanical properties, biodegradability and proliferation of osteoblasts on cement composites has been studied [5]. These systems were composed of calcium phosphates and a liquid phase ensuring sufficient viscosity, which, in addition, can react with the cement mixture or serve to polymerize with the aim to strengthen the overall composite system. Another possible solution is represented by the formation of composite hydrogel systems in combination with solid powdered cement mixtures of calcium phosphates, which affects their mechanical, physico-chemical and biological properties [6,7,8,9] Hydrogels in the systems mimic both the extracellular matrix and biomimetic structure of hard tissues, which consequently support the adsorption of specific growth factors, adherins as well as the interaction of the cells themselves [10]. On the other hand, the mechanical properties are generally significantly reduced but are suitable for the reconstruction of defects especially in the trabecular bone. From the point of view of the applicability of cements, an extreme increase in their setting time is not acceptable either. In the case of hybrid composites, the direct precipitation of calcium phosphates into neutral hydrogels, e.g., polyvinyl alcohol, hydroxymethyl cellulose [6,11] was applied and the stronger mutual interactions were observed using polyelectrolytes such as chitosan and gelatin [12,13]. To form specific calcium phosphate structures, hydrogels from collagen, silk, various peptides, soluble forms of cellulose, poly-lactic acid. chitosan or solid polymeric substrates have been extensively studied [14,15]. The mutual interactions between biopolymer polyelectrolytes with the formation of complexes [15,16] as well as their ability to form complexes with divalent and trivalent metal ions [17] are important for the strengthening of the microstructure of composites. The calcium phosphate-loaded carboxymethyl cellulose (CMC) nonwoven sheets showed a significantly higher new bone formation rate in the composites than that in the CMC group [18]. The CPC/poly(lactic-co-glycolic acid/CMC composite showed a good long-term biological response and the new bone formation in vivo [19]. The TTCP/DCPA/CMC cement had adequate osteoconductivity, biocompatibility and adequate compressive strength in an in vivo sheep vertebral bone void model [20]. The calcium phosphate composite cements with polyacrylic acid (PAA) demonstrated adjustable brittle/ductile strength at high PAA contents (up to 50 wt%) after setting [21].

The phytic acid (PHYT) had positive effects on depressing the stone formation, stem calcification, reducing the progression of osteoporosis and, e.g., an anticancer effect [22,23,24]. The PHYT can reduce the pH in the body defect site with a possible positive effect on the activity of osteoclasts (at weak acid or neutral pH (6.5–7) conditions) [25]. The formation of Ca^2+^-PHYT complexes is responsible for the decrease in the crystallization rate of hydroxyapatite [26]. Retarding properties of PHYT were demonstrated in the case of setting Mg-type cement with newberyite and farringtonite as final products [27]. In previous work, we studied the possibility of the preparation of TTCP/DCPA fast-setting cement with hardening liquid containing PHYT and the influence of PHYT on cement properties [28].

The above facts clearly indicate the relevance to focus on the preparation and development of new types of hybrid biocement self-setting systems with in situ precipitated calcium phosphate nanoparticles in biopolymer polyelectrolyte, which have been studied relatively little in this connection. In this paper, we focused on the preparation of hybrid injectable self-setting TTCP/DCPA cements with hardening liquid composed of the PHYT as organic phosphate source, the polymeric CMC/ PAA polyelectrolytes for improving injectability and the fungal phytase for partial enzyme degradation of PHYT which affects cement setting process. The selected anionic organic additives contain carboxylate groups which—similar to PHYT acid—can interact with calcium in CPC and influence the microstructure formation. Moreover, we assume no mutual electrostatic interactions between individual organic compounds in hardening liquid due to their negative charge from which the relative high long-term stability of hardening liquid results.

## 2. Materials and Methods

### 2.1. Preparation of Cement Mixtures and Cement Pastes

The tetracalcium phosphate/monetite powder mixture (TMPM) was prepared by milling of TTCP in diluted solution of orthophosphoric acid (86% analytical grade, Merck, Darmstadt, Germany) in 80% ethanol. The final Ca/P mole ratio in TMPM equal to 1.67 (C cement—stoichiometric hydroxyapatite). Reaction milling was done in the planetary ball mill with agate balls (3 balls, 1 cm in diameter) and vessel for 30 min. Tetracalcium phosphate (Ca_4_(PO_4_)_2_O, TTCP) was synthesized by annealing of an equimolar mixture of calcium carbonate (CaCO3, analytical grade, Sigma-Aldrich, Saint Louis, MO, USA ) and dicalcium phosphate anhydrous (DCPA) (CaHPO_4_ (Ph.Eur.), Fluka) at 1450 °C for 5 h with following milling in a planetary ball mill (Fritsch, 730 rpm, agate balls and vessel) for 2 h. The phase purity was analyzed using X-ray powder diffraction analysis (XRD, Philips X Pert Pro, Malvern Panalytical B.V., Eindhoven, The Netherlands).

The cement pastes (CXI) (Table 1) were prepared by the mixing of TMPM powder mixture with 2% solution of sodium carboxymethyl cellulose (CMC, Sigma-Aldrich, sodium salt, M_w_ = 250 kDa) containing 0.5 or 1% of polyacrylic acid (PAA, Sigma-Aldrich, M_w_ = 100 kDa, 35% solution.) and 8 wt% of phytic acid (PHYT) with addition of phytase (F3P, Natuphos^®^, BASF, Ludwigshafen, Germany). Two P/L ratios equal 1.7 and 2 were used in experiments. The PHYT and F3P were separately dissolved in CMC/PAA solutions and mutually mixed for 60 s. The final Ca/P molar ratio (including phosphates in PHYT) was 1.50. The PHYT/F3P mass ratio was 20. The F3P was extracted from NATUPHOS^©^ phytase product in distilled water and after precipitation with ammonium sulfate (50% solution), was filtered and dialyzed (Millipore dialyzer, cut off 8 kDa) against distilled water for 4 days. The phytase activity determined according to [29] was 108 U/mg of enzyme. 

### 2.2. Characterization Methods

#### 2.2.1. Injectability, pH Measurement and Release of Ions from Cements during Soaking 

The changes in pH of simulated body fluid (SBF) solution during soaking of 600 mg pellets (0.5 mm in thickness and 10 mm in diameter)/15 mL SBF after 24 h setting in 100% humidity at 37 °C were measured using a pH-meter (WTW, Inolab 720) with the SenTix41 combined electrode. The CXI pellet of 500 mg weight was added to 15 mL of 0.9% NaCl solution at 37 °C after 10 min setting in 100% humidity and slowly mixed in mini-rotator (BioRS24, Biosan, Riga, Latvia) at speed of 2 rpm. The total concentrations of released calcium and phosphate ions in NaCl solutions were analyzed using ICP (Horiba Activa) at selected soaking times (4, 24 h, 3, 7 days) and the free phosphate ions were determined by the colorimetry as P–Mo–V complexes [30]. The Ca/P ratio in CXI cements was determined by ICP after setting for 7 days in SBF solution at 37 °C and dissolution of samples in HNO_3_(analytical grade, 1 + 2).

The injectability of cements was tested by the extrusion of cement paste from polypropylene syringe. Briefly, a 10 mL syringe with the nozzle inner diameter of 2 mm was filled with cement pastes (approximately 5 mL) after mixing cement powder mixture with hardening liquid at a given P/L ratio. The syringe was fixed vertically in a holder and the paste was extruded from syringe after 3 min from addition of hardening liquid to cement using a universal testing machine (LR5K Plus, Lloyd Instruments Ltd., West Sussex, UK) at a crosshead speed of 10 mm/min up to achievement of 100 N load limit. The cement injectability % represents the relative mass amount of extruded paste to origin mass of cement paste in a syringe. The injectability was expressed as mean ± SD (n = 4).

#### 2.2.2. Compressive Strength, Phase Analysis, Setting Time and Microstructure of CXI Cements

The injectable CXI cement pastes were molded into the shape of a cube (10 × 10 × 10 mm) for measurement of compressive strength by packing in plastic 3D printed form. The pastes were introduced to a 5 mL syringe, injected to prepared plastic forms and hardened in 100% humidity at 37 °C for 30 min. The following samples were immersed in simulated body fluid (SBF) solution at pH = 7.4 and 37 °C and soaked for 7 days. The compressive strength (5 samples) was measured after demolding samples on a universal testing machine (LR5K Plus, Lloyd Instruments Ltd. West Sussex, UK) at a crosshead speed of 1 mm/min. The phase composition of samples was analyzed by X-ray diffraction analysis (Philips X’ PertPro, using Cu Kα radiation, Malvern Panalytical B.V., Eindhoven, Netherlands) and FTIR spectroscopy (Shimadzu, Kyoto, Japan, IRAffinity1, 400 mg KBr + 1 mg sample). 

The microstructure of cements was observed by field emission scanning electron microscopy (JEOL FE SEM JSM-7000F, Tokyo, Japan). The final setting time of cement pastes was evaluated according to ISO standard 1566 [31] (Vicat method). The porosity of samples was calculated from measured dimensions and weight of samples. The theoretical density of hydroxyapatite (3.15 g/cm^3^) was used for calculation.

#### 2.2.3. Cytotoxicity of Extracts, Viability Testing and ALP Activity of Osteoblasts, Live/Dead Staining

The CXI pastes were prepared with hardening liquid and embedded in a sterile 50 mL polypropylene centrifuge tube with the complete osteogenic differentiation culture medium (the α-modification Minimum essential medium Eagle (10% FBS with osteogenic supplements L—ascorbic acid 50 µg/mL, 50 nM Dexametasone, 10 mM β-glycerophosphate and 1 % penicillin, streptomycin, amphotericin (Sigma-Aldrich)) in a ratio of 0.2 g cement powder per mL of medium (A) (in accordance with ISO 10993-12:2012 [32]) and 0.1 g cement/mL medium (B1) in the case of long-term testing (9 and 15 days). The CXI pastes samples were prepared also in basic medium EMEM without osteogenic supplements according to the same method described above for long-term testing (B2, 0.1 g/mL).

Samples were soaked in medium in an incubator at 37 °C for 24 h and subsequently, the cytotoxicity of A extracts was tested according to ISO 10993-5:2009 [33]. MC3T3E1 cells were harvested from culture flasks by enzymatic digestion and resuspended in culture medium. The cell suspension was adjusted at a density of 1.0 × 10^5^ cells/mL. Briefly, 1.0 × 10^4^ of pre-osteoblastic MC3T3E1 (Subclone 4) cells (ATCC CRL- 2593, Manassas, VA, USA) cells were suspended in 100 µL of EMEM + 10% FBS, 1% antibiotic solution and seeded into each well of the 96-well cell Grade Brand microplate (adherent wells) and cultured to a semi-confluent monolayer at 37 °C, 95% humidity, and 5% CO_2_ in an incubator for 24 h. Subsequently, the culture medium in wells was replaced with 100 µL of 100% extract (A, B1 and B2). All experiments were carried out in triplicate and cells in wells with extract-free complete culture medium were considered as a negative control. After 24 h of culturing, the A extracts were replaced with fresh culture medium and the in vitro cytotoxicity was evaluated by the MTS proliferation test assay (Cell titer 96 aqueous one solution cell proliferation assay, Promega, Promega Corporation, Madison, WI, USA). The absorbance of formazan was determined by a UV-VIS spectrophotometer (Shimadzu). The B1 and B2 extracts were used for long time osteoblast viability testing where culture extracts were exchanged every two days.

The CXI pastes for contact cytotoxicity assessment were prepared with hardening liquid and molded to a disc form (10 mm in diameter, 0.5 mm in height) and hardened in 100% humidity at 37 °C for 24 h in an incubator. Samples were sterilized three times at 105 °C for 45 min. In total, 2 × 10^4^ pre-osteoblastic MC3T3E1 (Subclone 4 cells) (ATCC CRL- 2593, Manassas, VA, USA) were seeded in 0.4 mL of culture medium (EMEM, 10 % FBS) on cement surfaces in 48 well culture plates and cultured at 37 °C in CO_2_ incubator.

The ALP activity of osteoblasts was determined in cell lysates after lysis in solution containing 0.1% Triton X-100, 1 mM MgCl_2_ a 20 mM Tris. The cell lysates were transferred into 1.5 mL microcentrifuge polypropylene tubes, frozen at −20 °C and centrifuged at 10,000 rpm for 10 min after thawing. The 100 µL of cell supernatant was added to 100 µL of p-nitrophenyl phosphate in diethanolamine buffer (0.5 mM MgCl_2_, pH 9.8) and incubated at 37 °C. The reaction was stopped after 60 min with 50 µL of 3 M NaOH. The amount of p-nitrophenol produced during the ALP enzyme catalysis of the p-nitrophenyl phosphate substrate was determined from the calibration curve of p-nitrophenol at 405 nm using the UV VIS spectrophotometer. The ALP activities were expressed in nanomoles of the p-nitrophenol produced per 1 min per microgram of proteins. The content of proteins in lysates was evaluated by Bradford´s method with Coomasie blue G250 as the complexing agent [34]. The statistical evaluation of results (n = 4) was performed using one-way and two-way ANOVA analysis at level α = 0.05 (Statmost program). 

The distribution and morphology of cells cultured in tested extracts and on discs were visualized with live/dead staining (fluorescein diacetate/propidium iodide) by fluorescent microscopy (Leica DM IL LED, Heerbrugg, Switzerland). 

Alizarin red S staining of deposits after culture of osteoblasts in cement extracts was carried out after washing wells with PBS, fixing in ethanol for 10 min and final double washing with deionized H₂O. Deposits were stained with Alizarin red S staining solution for 30 min. After removal of staining, solution wells were washed four times with water and observed under light microscope (Leica DM IL LED). Calcium deposits produced by cells were stained bright red. For quantitative analysis of deposits production by osteoblasts in different extracts, the calcium concentration in wells after the Alizarin red staining was determined by ICP. Calcium deposits were dissolved in HNO₃ (1 + 2, analytical grade, Sigma-Aldrich) before analysis. 

#### 2.2.4. Gene Expression

For the extraction of total RNA we used approximately 1 × 10^6^ cells. Total RNA from each cell culture was extracted using the RNeasy Mini Kit (Qiagen, Germantown, MD, USA) following the manufacturer’s instructions. Contaminating genomic DNA was digested using the RNase-free DNase set (Qiagen, Germantown, MD, USA). The RNA quality and yields were analyzed using the Nanodrop spectrophotometer (Thermo Scientific, Waltham, MA, USA). Complementary DNA (cDNA) synthesis was performed using the protocol for RT2 First Strand Kit (Qiagen, Germantown, MD, USA), where 1 µg of total RNA was used (after the genomic DNA elimination step) to prepare 20 µL of cDNA. cDNA was then used for real-time PCR experiments.

The quantification of genes of interest in the cDNA samples was performed using primers for IGF1, osteocalcin and osteopontin (Table 2). 

A 25 µL reaction mixture, each consisting of triplicate samples of cDNA, specific primer mix and RT2 SYBR Green qPCR mastermix (Qiagen, Germantown, MD, USA) was set up in each well of a 96 reaction plate (Roche, Switzerland). cDNA for β-actin was used as endogenous control for calculating fold differences in RNA levels of cells treated vs. not treated by biomaterials by the 2-ΔΔCT method. The plate was sealed using optical adhesive cover (Roche, Switzerland) and was placed in LightCycler 480 II real-time PCR system machine (Roche, Switzerland). The real-time PCR was performed under the following conditions: initial incubation at 95 °C for 10 min, amplification in 45 cycles at 95 °C for 15 s followed by 60 °C for 1 min. Amplification specificity was checked by the generation of a melting curve. The statistical evaluation of results was performed using two-way ANOVA analysis at level α = 0.05 (Statmost program).

## 3. Results 

### 3.1. XRD and FTIR Analysis

**Figure 1:**
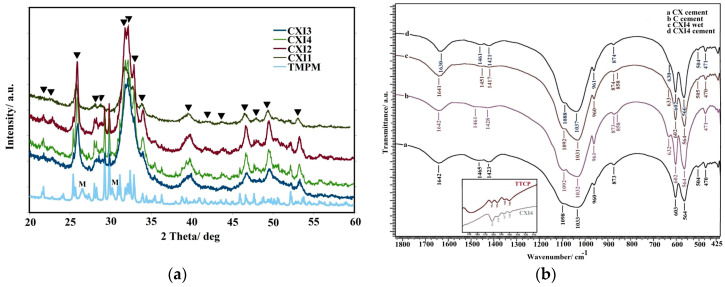
X-ray powder diffraction (XRD) patterns of CXI cements after 7 days soaking at 37 °C in simulated body fluid (SBF) (M-monetite, ▼ HAP) (**a**) and FTIR spectra of C cement using 2% NaH_2_PO_4_ hardening liquid, CX cement after setting in PBS at 37 °C for 7 days; CXI4 cements setting in 100% humidity and soaking in PBS solution at 37 °C for 24 h and 7 days respectively (**b**).

In Figure 1a, the XRD patterns of CXI cements after 7 days soaking at 37 °C in SBF are shown. The final product of calcium phosphate cement transformation was calcium deficient nanocrystalline hydroxyapatite with a small carbonate substitution (HAP) (ICDD standard PDF4 01-071-5048). In CXI cements, a strong reduction in the amount of untransformed TTCP phase (JCPDS 25-1137) originated from starting cement mixtures (from about 23 to 7% and from 12% to traces of origin TTCP amount in cement with 1% of PAA and 0.5% of PAA respectively) with decreasing P/L was demonstrated. Moreover, the remains of monetite phase (lines from reflections of (020), (−220) and (−112) monetite planes, JCPDS09-0080) were clearly visible in CXI cement with 1% PAA. The crystallinity sizes in CXI cements calculated from the Scherer’s equation (from the reflection of (002) plane of HAP) were close to 38 nm except for CXI1 cement where the crystallinity size was around 24 nm. No additional lines from any secondary phases were identified in patterns. The formation of calcium-deficient hydroxyapatite also verified chemical analysis of CXI cement after 24 h setting in SBF, where the Ca/P_free_ ratio and total Ca/P ratio were 1.60 ± 0.04 and 1.54 ± 0.02 respectively. 

FTIR spectra of C cement using 2% NaH_2_PO_4_ hardening liquid, CX cement after setting in PBS at 37 °C for 7 days; CXI4 cements setting in 100% humidity and soaking in PBS solution at 37 °C for 24 h and 7 days respectively are shown in Figure 1b. In the IR spectra of all cements, antisymmetric (ν_3_) and symmetric (ν_1_) P–O stretching vibrations of PO_4_^3−^ group in hydroxyapatite located at 1090, 1033 and 962 cm^−1^, O–P–O bending (ν_4_) vibrations at 565 and 602 cm^−1^ and librational mode of OH group in HAP around 630 cm^−1^ were found [37,38]. Bands characteristic for ν_3_ and ν_2_ vibrations of CO_3_^2−^ group in carbonated HAP at about 1460, 1420 and 870 cm^−1^ represent the B-type CO_3_^2−^ substitution for PO_4_^3−^ groups in HAP [39]. The low intense peak from stretching vibrations of the OH group in hydroxyapatite was identified at around 3570 cm^−1^. The peak at about 860 cm^−1^ found in the spectrum of CXI4 cement arises from vibrations of the hydrogen phosphate group in HAP [39,40] or a labile carbonate environment [41]. Besides the comparison of FTIR TTCP bands between 900 and 980 cm^−1^ with the same bands of deconvoluted CXI spectrum, peaks at 940, 947, 955 and 962 cm^−1^ from ν_1_ symmetric stretching vibration of PO_4_ group in TTCP [42] were clearly identified in spectrum. This fact is in accordance with the XRD analysis of CXI patterns.

No stronger FTIR bands in spectra of CXI cements corresponding to Ca-PHYT complexes at 1134, 996 and 842 cm^−1^ [43] were found after soaking in PBS solution or hardening in 100% humidity at 37 °C. 

### 3.2. Microstructure and Particle Morphology in CXI Cements

**Figure 2:**
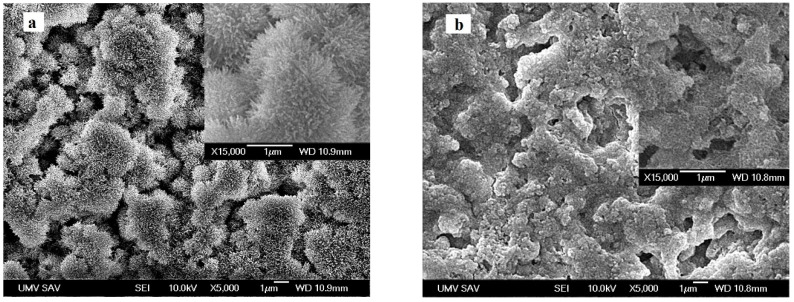

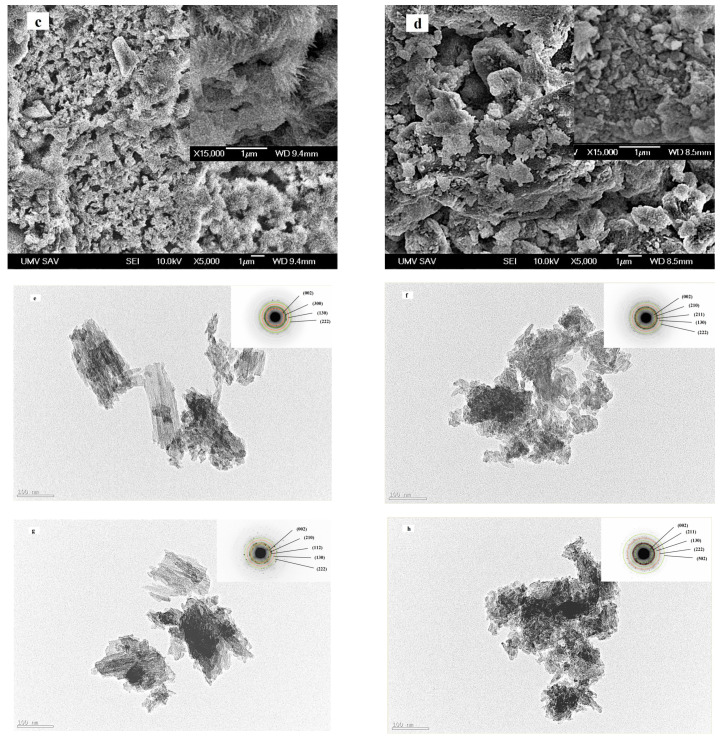
Microstructure of CXI cements and particle morphology after 7 days setting in SBF solution at 37 °C (**a**,**e**—CXI1, **b**,**f**—CXI2, **c**,**g**—CXI3, **d**,**h**—CXI4).

The microstructure of CXI cements, as well as more detailed images of particle morphology after 7 days of setting cements in SBF solution at 37 °C, are shown in Figure 2. From the comparison, larger differences in HAP particle morphologies can be seen with the change in P/L ratio than in the content of PAA in CXI cements, which are visible in images. Thus, the higher portion of needle-like hydroxyapatite particles can be seen in CXI cements at P/L = 1.7 (Figure 2a,c) contrary to cements at P/L = 2 where very fine mainly globular particles were found (Figure 2b,d). The needle-like HAP nanoparticles were joined in the form of spherical agglomerates about 1–5 µm size in CXI1 cement whereas the mixture of irregularly shaped strongly microporous agglomerates (around 1 µm size) composed of fine globular nanoparticles (<100 nm size) and thin needle-like submicron particles were revealed in the CXI3 sample by SEM. Note that the individual agglomerates were partially separated by a gap of micropores (up to 2 µm) in the CXI1 sample while such a boundary was significantly thinner (<1 µm) and not so sharply distinguished in CXI3. The irregularly shaped micropores had the length of a few micrometers. The TEM images clearly verified observations from the SEM analysis, the length of needle-like HAP particles were up to 200 nm and 100 nm (thickness around 20 nm in both cements) for CXI1 and CXI3 samples respectively. Moreover, very fine 10 nm globular particles joined to agglomerates and mixed with needle-like particles were identified in TEM images of both cements too (Figure 2, for example). 

In the case of CXI cements with P/L = 2, the hydroxyapatite agglomerates of 5–10 µm size composed of submicrometric globular particles were identified in microstructures (Figure 2b,d). A relatively high fraction of irregularly shaped micropores of up to 10 µm size was revealed in both cements but no clearly visible separation of agglomerates was visible in images from which resulted that a more compact microstructure was formed during the hardening of cements. If we compared the morphology of HAP particles observed using TEM, the significant particle refinement was found in 1%PAA CXI4 cement where the diameter of spherical particles did not exceed 10 nm contrary to the dimension of particles in the form of rods (up to 50 nm) in CXI2 sample (Figure 2f,h). Rods were mutually mixed with a lower portion of the finer spherical nanoparticles in CXI2 cement. Note that the SAED (selected area electron diffraction) analysis confirmed the formation of hydroxyapatite particles in all hardened cements.

Results from the relative density analysis (RD) of hardened cements clearly showed the rise in RD from 44% to 49% with the P/L ratio while the content of PAA in cements had an insignificant effect on RD.

### 3.3. Analysis of Ion Release, pH Measurement, Compressive Strength, Setting Time and Injectability

**Figure 3:**
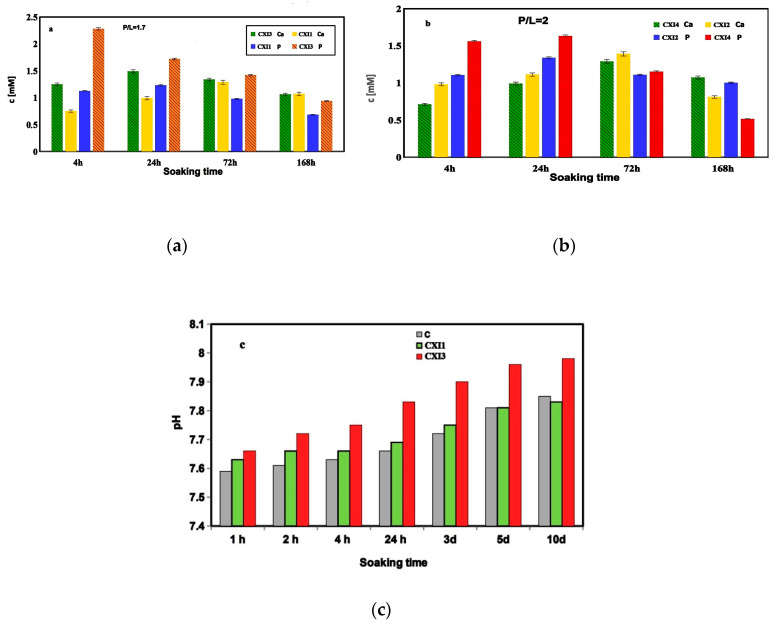
Release of calcium and phosphate ions (**a**,**b**) and change in pH values (**c**) with soaking time of CXI cements in 0.9 % NaCl and SBF solutions respectively.

The release of calcium and phosphate ions and the change in pH values with soaking time of CXI cements in 0.9% NaCl and SBF solutions respectively are depicted in Figure 3. Much higher concentrations of Ca and phosphorus were verified in NaCl solution with 1%PAA cement at the P/L ratio = 2 as compared with these ones at P/L = 1.7 while only insignificant differences in concentration of both species with the P/L ratio were identified in NaCl solution containing 0.5% cements. On the other side, the release of elements depended on the content of PAA in cements, especially during the first 24 h of soaking where the concentration of Ca and P was significantly higher in solutions with 1% PAA cements at P/L = 2 as well as an enhanced content of P was found in solution with 1 % PAA cement at P/L = 1.7 (statistically significant difference, *p* < 0.05) (Figure 3a,b). The Ca concentration achieved maximum after 72 h soaking CXI cements at P/L = 2 and gradually decreased whereas the P concentration gradually decreased after 24 h soaking cements at P/L = 1.7. Simultaneously with the above-presented changes, a slow increase in pH up to 8 was observed at the time period when the Ca^2+^ concentration rose contrary to the P concentration (Figure 3c). 

The wet CS of CXI cement after setting in SBF for 7 days was influenced by the P/L ratio, where the CS of 7 ± 2 MPa and 15 ± 3 MPa was measured for the P/L equal 1.7 and 2 respectively. None effect of the PAA content in cement on wet CS was identified. Similarly, the setting time of CXI cements was reduced from 28 ± 1 min to 7 ± 1 min with the rise in P/L ratio from 1.7 to 2. ST can be significantly reduced by the prolongation of the reaction time between PHYT and F3P during mixing of hardening liquid, e.g., ST of CXI3 cement fell down from 28 to 8 min for reaction times 30 s and 1 min respectively. Dry CS′s of cements were around 22 ± 3 and 21 ± 2 MPa in CXI1 and CXI3 samples respectively, and rose in CXI2 and CXI4 cements to 29 ± 4 and 33 ± 2 MPa respectively at higher P/L. Note that all CXI cements were fully injectable regardless of the P/L ratio but a more viscously paste was formed at the P/L = 2. 

### 3.4. Evaluation of In Vitro Cytotoxicity of Extracts, Live/Dead Staining and Production of Ca Deposits by Osteoblasts in Extracts, Gene Expression of Markers 

**Figure 4:**
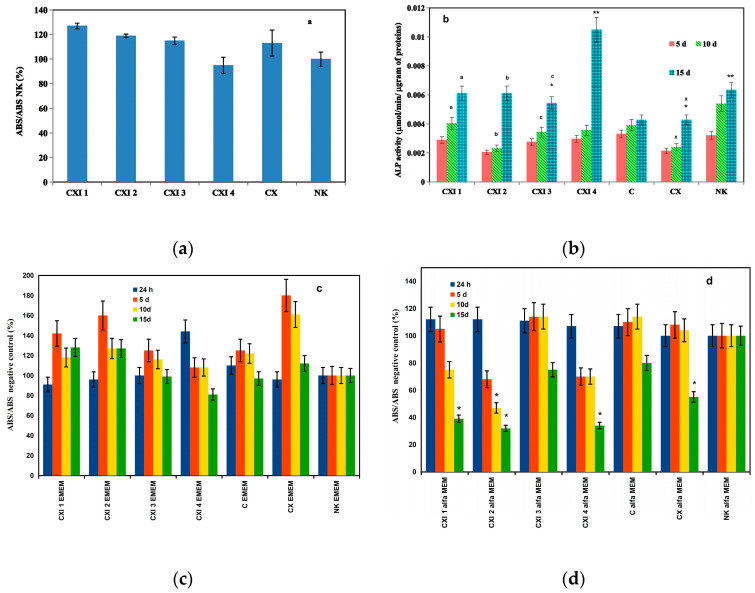
Relative viability of cells cultured in 100% cement extracts (according to ISO 10993-12:2012) (**a**); ALP activity of osteoblasts cultured in 50% αMEM CXI extracts (**b**) (statistically significant difference: a (*p* < 0.006), b (*p* < 0.0005), c (*p* < 0.01), x (*p* < 0.05), * (*p* < 0.015), ** (*p* < 0.0002)) and dependence of relative viabitily of osteoblasts on culture time in 50% EMEM (**c**) and αMEM (**d**) cement extracts (* under 70% non-cytotoxic level of control, *p* < 0.05).

**Figure 5:**
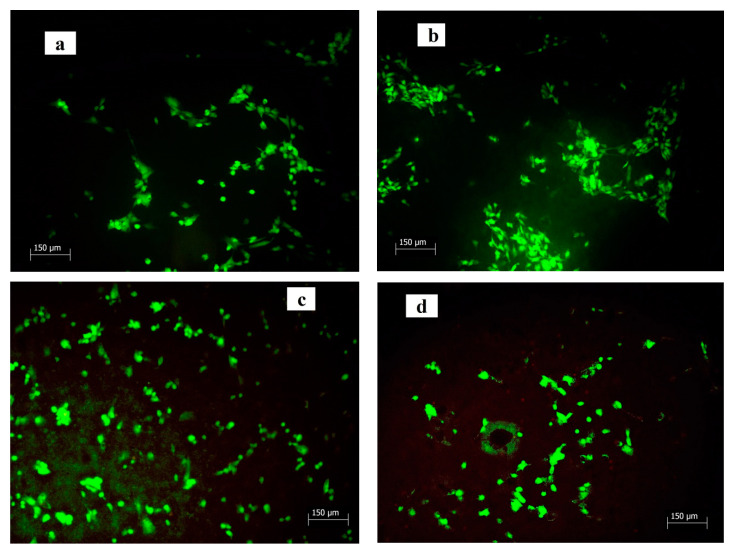
Morphology of cells cultured on cement surfaces for 48 h in αMEM at 37 °C (live/dead staining; **a**—C cement, **b**—CX cement, **c**—CXI1 cement, **d**—CXI3 cement; magnification: 100×).

The in vitro cytotoxicity testing of 100% cement extracts (A) (according to ISO 10993-12:2012) clearly confirmed the noncytotoxic character of extracts (Figure 4a). No differences in the morphology of cells cultured on cement surfaces for 48 h in αMEM were identified by live/dead staining (Figure 5) and on images are only visible live cells of globular poorly prolonged morphology adhered to cement surfaces. For the long-term cytotoxicity testing, 50% cement extracts (0.1 g cement/mL medium) in the osteogenic αMEM medium (B1) and MEM medium (B2) without any supplements were used for the culture of osteoblasts (we assume a relatively high dilution of species released from cements to body fluids after implantation).

Dependences of the relative viability of osteoblasts on culture time in 50% extracts (Figure 4c,d) revealed high viability of osteoblasts in the EMEM extracts free of osteogenic supplements regardless of the culture time contrary to viability in the osteogenic αMEM extracts where it was strongly reduced after 10 days of culture. After 15 days of culture in αMEM CXI extracts, the viability of osteoblasts decreased under 70% non-cytotoxic level (according to ISO). From the comparison of ALP activities, it resulted that the ALP activity of osteoblasts in 50% αMEM CXI extracts statistically significantly rose (*p* < 0.05) after 15 days of culture and it was significantly higher than the ALP activity of osteoblasts in the case of CX or C extracts (Figure 4d). 

**Figure 6:**
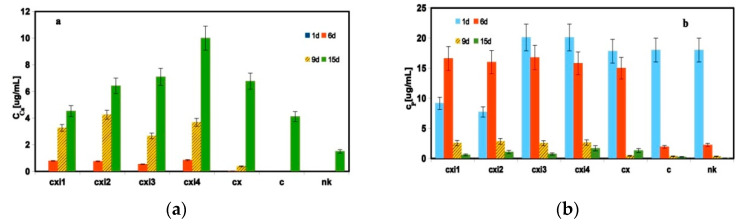

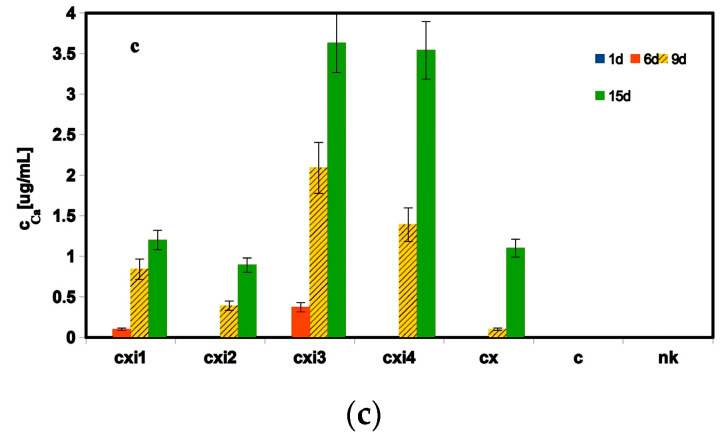
Concentration of calcium and phosphorus in deposits produced by osteoblasts cultured in wells for selected times in 50% αMEM (**a**,**b**) and EMEM (**c**) CXI extracts.

For the evaluation of the number of calcium deposits produced by osteoblasts in extracts, the concentration of calcium and phosphorus at selected culture times was determined after the dissolution of deposits in wells. While the concentration of calcium in wells increased with culture time in 50% αMEM cement extracts especially after 6 days of culture (Figure 6a), the content of phosphorus was very high during the first 6 days of cultivation in CXI extracts (Figure 6b). In the C extract and NK, the amount of phosphorus rapidly fell down (almost 10 times) even after 6 days of cultivation (Figure 6b). This tendency was observed in CXI extracts after 9 days of culture. The enhanced concentration of calcium was also measured in CXI EMEM extracts but the Ca concentration was much lower than in the αMEM extracts (3–10 times) (Figure 6c) as well as a very low amount of calcium deposits was produced by osteoblasts in the C extract. Note that the traces of phosphorus were identified in EMEM extracts (<0.1 mM) by ICP only. 

**Figure 7:**
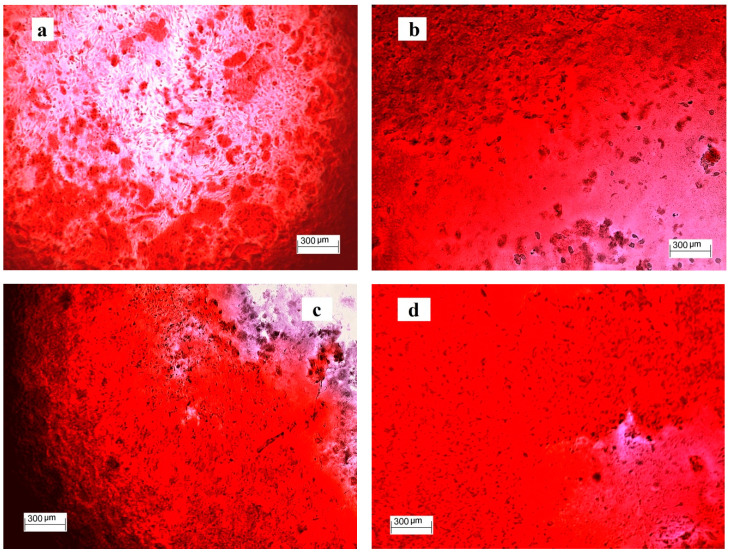
Formation of calcium deposits in wells after 15 days of culture of osteoblasts in 50% αMEM cement extracts (alizarin red staining; **a**—C, **b**—CX, **c**—CXI1, **d**—CXI3).

**Figure 8:**
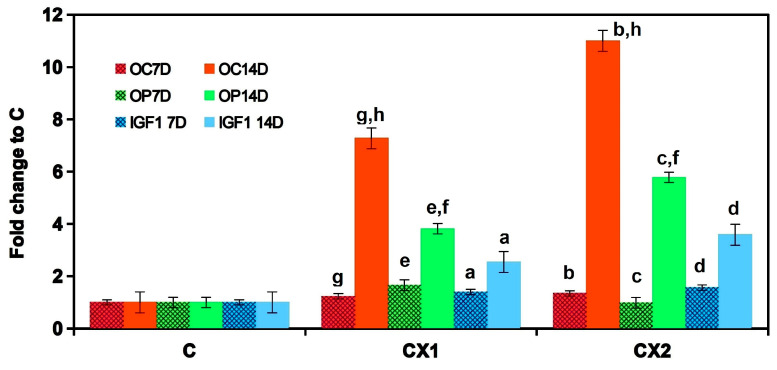
Relative gene expression from RT-qPCR analysis of cell lysates cultured for 7 and 14 days (statistically significant difference, *p* < 0.01)).

The formation of a large number of calcium deposits in 50% αMEM cement extracts is demonstrated by images after alizarin red staining (Figure 7). The deposits in wells were intensively red-stained due to the interaction of the dye with calcium deposits in CXI extracts after 15 days of cultivation. This result is in accordance with the rise in both the Ca content and ALP activity in CXI wells. It is clear from the comparison of the above facts that PHYT supported the formation of Ca deposits but the additional synergistic effect of PAA and CMC on enhanced ALP activity of CXI cements was verified. For verification of osteoblastic activity of cells, the osteogenic gene expressions for osteopontin, osteocalcin and IGF1 growth factor were evaluated by the RT-qPCR analysis. The relative gene expression (Figure 8) clearly demonstrated the strong up-regulation of IGF1, OC and OP in osteoblasts cultured for 14 days in αMEM 50% CXI extracts (statistically significant difference) as compared with C samples and 7 days cultured cells in CXI cement extracts. 

## 4. Discussion

Based on chemical analysis and taking into account phosphates from PHYT or inositol phosphates in cements, the total Ca/P ratio in CXI cement was close to the theoretical, and no soluble Ca complexes after interaction with inositol phosphates (with any numbers of phosphate groups) that remained were released from cements. Despite the fact that approximately 8 wt% of PHYT was added to cement, the intensity of phytate peaks in FTIR spectra of CXI cements was very weak and we believe that the intensity of peaks was lowered due to the delocalization of electrons after bonding with surface calcium ions. Note that an amount of the admixed CMC and PAA was very low for their identification in CXI spectra but no mutual interaction including the PHYT is possible because of the dissociation of carboxylic and phosphate groups in compounds with following net negative charge at pH > 7. Similar types of organic complexes are known, e.g., between chitosan and PAA or PHYT but these electrostatic interactions were the result of the opposite charge density of reactants [44,45].

It was revealed that the calcium phosphate mineralization and the particle morphology of carboxymethyl cellulose-grafted polymethacrylic acid templates can be affected by the preparation conditions [46,47]. In the (hydroxypropyl)cellulose or (ethyl)cellulose/PAA composite mineralized films, three various calcium phosphate forms—the amorphous calcium phosphate, hydroxyapatite, and the PAA-Ca^2+^ complex—were identified after incorporation of the calcium and phosphate ions inside the polymer matrices from soaking solution [48]. On the other hand, a strong inhibition effect of the PAA on apatite formation was found in bioactive glass-ionomer cements, and the presence of a small quantity of PAA in SBF hindered the apatite formation on AW glass ceramics [49]. Similar to the case of CXI cements, it was identified that the remains of TTCP and DCPA phases were found in XRD patterns of the TTCP/DCPA cement with 2% addition of CMC gel but a small difference was only verified in comparison with XRD patterns of pure cement [50]. These facts indicate that the polymeric additives create complexes on the surface of calcium phosphate particles which hinder the complete transformation of CXI cements. Simulations of HAP-polymer interfacial molecular interactions demonstrated that the most favorable orientation of PAA for the interaction with HAP is along the c-axis of HAP aligned parallel to polymer chains for in situ HAP, contrary to ex situ HAP/PAA composites where the (001) HAP surface is the most probable plane for adsorption of PAA molecules [51]. The change in HAP particle morphology from rod-like to spherical as well as the structural change from nanocrystalline to amorphous phase was observed with increasing content of the PAA from 0.3 to 0.5 mg/mL during precipitation [52]. Thus, the refinement of HAP particles at a higher PAA content in CXI cements as well as the rise of the spherical fraction at the expense of rod-like particles can be associated with the change in the type of PAA adsorption in relation to HAP planes. It can be assumed that while the (100) plane was preferable for PAA adsorption on 0.5 PAA CXI cement particles, which inhibits the growth of HAP in this plane and simultaneously supports the growth of HAP particles in (001) plane and the formation of needle-like particles in CXI microstructures, the PAA adsorption in (001) HAP plane or even in both planes was enhanced at 1 wt% PAA content in cements with the formation of very fine spherical particles. Moreover, the growth of HAP particles was influenced by the content of liquid phase in CXI cement microstructure where a lower content of liquid (P/L = 2) caused faster achievement of supersaturation and shortening of diffusion distances between individual cement particles with the acceleration of precipitation on nucleation sites and following re-crystallization. These facts are important, especially in the case of viscous liquids with lower values of diffusion coefficients of ions in CXI cements containing CMC where higher contents of liquid are responsible for the existence of a large number of HAP nuclei which are stabilized in colloidal state and creates globular very fine particles or agglomerates like in CXI2 and CXI4 cements. Similar stabilization of colloidal HAP particles was revealed during the precipitation in presence of CMC [53,54].

From previous work, we know that the formation of Ca-inositol phosphate complexes as well as the presence of free phosphates affects the growth of HAP particles into the form of columnar thin individually separated HAP particles, forming wells around hollows in microstructure [28]. No such morphological type of particles was documented in the microstructure of CXI cements which clearly demonstrates the strong influence of polymeric additives on particle growth and formation of CXI cement microstructure. On the other hand, the significant effect of PAA concentration was demonstrated during precipitation of HAP where a rise in PAA concentration from 0.3 to 0.5 mg/mL caused the structural change from nanocrystalline to amorphous phase [52].

(1) $$\begin{array}{ll} {3\mathrm{Ca}}_{4}{\left({\mathrm{PO}}_{4}\right)}_{2}{O\;+\;3H}_{2}O\;\to & \underline{{2\mathrm{Ca}}^{2+}{+4\mathrm{OH}}^{-}}{+\;2\mathrm{Ca}}_{5}{\left({\mathrm{PO}}_{4}\right)}_{3}\mathrm{OH}\\ & \;\;\;↓\leftarrow {\;+\;\mathrm{PO}}_{4}{}^{3-}\left(\mathrm{from}\;\mathrm{PHYT}\right) \end{array}$$

3CaHPO_4_ +H_2_O → 3Ca^2+^ + 3H_3_O^+^ + 3PO_4_^3−^ (2)

↓

Ca_5_(PO_4_)_3_OH + H_2_O (3)

The comparison of results from the chemical analysis revealed that the amount of released P correlated with the content of PAA and PHYT in cements during the first stage of cement transformation (due to a higher addition of hardening liquid to cement (lower P/L ratio)) but no statistically significant difference (*p* < 0.05) was identified between the concentration of free phosphate ions and total phosphates in solutions at the selected time. Thus, it can be assumed that more stable insoluble PHYT complexes were created on the surface of origin calcium phosphate particles but the local acid environment due to acid hardening liquid supported an enhanced solubility and hydrolysis of the origin calcium phosphate mixture as well as prolonged the enzyme activity of F3P with releasing phosphate ions from PHYT which are consumed during the formation of hydroxyapatite nanoparticles in the later stage of the cement transformation. From the point of view of practical applications, it is an interesting fact that the pH changes in CXI SBF solutions with soaking time were relatively slow after 1 h from the cement immersion with a maximum not exceeding 8 for CXI3 cement. The lower pH values found in SBF of CXI1 cement as compared to CXI3 can be related to the faster achievement of supersaturation in relation to hydroxyapatite in CXI3 solution due to stronger hydrolysis of TTCP with the following interaction with monetite according to Equations (1)–(3). From the equarions, it is clear that the result is that hydroxyl ions are gradually consumed with both the prolongation of cement transformation and the pH solution rise. The enhanced TTCP hydrolysis was supported by a higher concentration of phosphates produced in more acidic conditions in CXI3 cement due to 1% PAA content and deeper enzymatic attack of PHYT with phytase. The phosphate ions shift the chemical equilibrium to the product side due to the consumption of calcium ions which arise from TTCP hydrolysis (Equation (1)).

The dependence of cement paste fluidity vs. the PAA/cement ratio was measured as a minimum but the high fluid paste was produced in an interesting region with <5% PAA due to the adsorption of PAA on the surface of cement particles [55]. It was shown that the setting time of TTCP/DCPA cements with the PAA content between 10–25% decreased with the PAA molecular weight to around 10 min for M_w_~100 kDa. Similarly, the CS of cements containing a high molecular PAA was reduced in comparison with the standard cement and achieved about 20 MPa for 10 wt% PAA cement (100 kDa) [56]. The significant influence of TTCP/DCPA ratio on the CS of PAA cements due to cross-linking PAA via Ca ions was measured [57]. On the other side, the rise in addition of CMC to the brushite/vaterite cement up to 50 wt% caused the increase in dry composite CS to 86 MPa as a result of cement porosity reduction [58]. The injectability (and cohesion) of αTCP cement was improved by 1.5 wt% addition of CMC up to 90% while the setting time decreased from 11 to 7 min [59]. Contrary to the above facts, the injectability of αTCP cements with admixing powder CMC was even reduced at lower contents of CMC (<1 wt%) as compared with the pure αTCP cement due to the segregation of the solid phase from paste during extrusion but rose at higher CMC contents (twice than pure cement). Moreover, the insignificant effect of the CMC addition on pH was observed [60]. The higher CS (around 60 MPa) was found in TTCP/DCPA composite cement with the high PAA content (up to 25 wt%) and simultaneous addition of tartaric acid/CaF_2_ mixture but the ductile cements were prepared at 50 wt% content of PAA due to particle coating with polymer and the formation of brushite [21]. Setting times were controlled by the P/L ratio in this case. A gradual transformation of αTCP cement to hydroxyapatite after mixing with concentrated PAA/2% Na_2_HPO_4_ hardening solution was identified but the setting time was around 22 min. CS and porosity of this composite cement did not exceed 22 MPa and 30% respectively [61]. It was demonstrated that a 2% addition of CMC to TTCP/DCPA cement in gel form caused the fast cement resistance to washout (<2 min) but the CMC addition had no effect on the CS of cement composite (around 45 MPa) at P/L = 3.75 [50]. In the case of CPC based on dicalcium phosphate with an addition of 5–20 wt% PHYT as hardening liquid, the CS achieved about 16 MPa and the PHYT caused injectability of cements due to the retardation of the setting process [62]. On the other hand, cement powder in the form of HAP particles coated with PHYT, which was hardened with water, had the CS maximal of 10 MPa [63]. 

From the comparison of results with the above papers, the CXI cements are characterized by similar or improved properties than in presented cements as, for example, the almost complete transformation to hydroxyapatite, the full injectability with the high resistance to washout in aqueous solutions in a short time after mixing, the sufficient value of CS comparable with bone tissue as well as the easily controllable setting process via interaction between PHYT and F3P whereas much lower amounts of organic poly-electrolytes were admixed to cements.

CXI extracts stimulated up-regulation in the expression of osteogenic genes. The high gene over-expression, especially OC (up to 11-fold) and OP (6-fold), relative to C extract corresponds with the large number of calcium deposits produced by osteoblasts in CXI extracts, thus, additives in CXI cements significantly supported the maturation and activity of osteoblasts. It is known that the IGF1 factor stimulates the type I collagen synthesis, osteocalcin production and alkaline phosphatase activity [64,65]. On other hand, the osteocalcin (OC) has been found to accelerate nucleation by promoting the growth of nanosized calcium phosphate ion clusters and suppressing hydroxyapatite crystal growth [66,67] as well as osteopontin (OP) in mixture with osteocalcin-promoted hydroxyapatite formation [68]. Both proteins are induced in the mineralization stage and are characterized by mature osteoblasts [69]. Calcium phosphate-loaded CMC sheets promoted calcification and marker gene expressions, ALP activity for later phase of osteoblast differentiation as well as supported a new in vivo bone formation in a mouse calvaria defect model [70]. Hydroxyapatite/CMC hybrid hydrogel composites showed good adhesion, cell viability as well as osteogenic and odontogenic differentiation of dental pulp stem cells contrary to CMC hydrogel with poor cell adhesion [71]. There was no cytotoxicity. cell adhesion, proliferation and enhanced osteogenic activity of cells (alkaline phosphatase activity, expression of osteogenic marker genes and mineralization) were identified on the 4-layer poly(allylamine hydrochloride)/PAA films but the mechanical properties of films may play a critical role in modulating the behavior of osteoblasts [72]. The effects of PHYT on the activity of osteoblasts were evidenced by the enhanced production of osteopontin which regulates the growth of hydroxyapatite particles without any adverse influence on proliferation, ALP activity or collagen production. It was revealed that the micromolar amounts of PHYT added to the osteogenic medium significantly reduced mineralization without affecting cell viability, proliferation or collagen deposition by osteoblasts contrary to the insignificant effect of lower inositol phosphate [73]. This fact clearly supports our findings because more than 25% of phosphates in PHYT were released into the hardening liquid due to enzyme hydrolysis with phytase in CXI cements, thus, the majority of PHYT was present in the form of lower inositol phosphates even if the micromolar amount of inositols was desorbed from CXI surfaces neither could it affect on cell viability. In the above study, suppressing the proliferation of osteoblasts was observed in nonosteogenic medium with PHYT addition (micromolar) after long-term cultivation. CPC composed of TTCPM and 10% PAA hardening liquid showed moderate osteoblast cytotoxicity after the first 24 h but the cytotoxicity decreased with culture time which was related to the possible release of unreacted PAA and lower pH value, thus the addition of a much higher concentration of PAA had a relatively weak effect on the long term cytotoxicity of cements [74]. Similar to CXI cements, the ALP activity of bone marrow stem cells rose after 14 days of culture as compared to 7 days as it was demonstrated in the case of hydroxyapatite cements with the lower addition of PAA (up to 2 wt%) and no influence on the viability of cells was shown which confirms the insignificant effect of low PAA contents on in vitro cytotoxicity of CPC [75]. Note that the complex polyelectrolyte composition of hardening liquid was responsible for good bioactivity of noncytotoxic CXI cement extracts in relation to the production of calcium deposits, ALP activity and expression of osteogenic markers by osteoblasts. 

## 5. Conclusions

The new enzymatically hardened hybrid tetracalcium phosphate/monetite cements with the small addition (up to 1 wt%) of anionic PAA/CMC polyelectrolyte and hardening liquid containing PHYT/ phytase mixture were fully injectable and self-setting. The results showed the high resistance to wash-out in a short time after cement mixing with hardening liquid. The simple control of setting time (7–28 min) was revealed via the change in P/L ratio in cement paste or reaction time between PHYT and phytase. The larger differences in HAP particle morphologies with the change in P/L ratio than in the content of PAA were identified in CXI cements where the higher fraction of needle-like and fine spherical HAP nanoparticles were found at P/L ratio =1.7 and 2 respectively. The wet CS′s of CXI cements (up to 15 MPa) were comparable with cancellous bone. XRD analysis verified the fast transformation of cements to hydroxyapatite but the enhanced remains of origin calcium phosphates in hardened CXI cements were shown at higher P/L ratio and 1%PAA content. The analysis of in vitro cytotoxicity confirmed the noncytotoxic character of cement extracts and the RT-qPCR osteogenic gene analysis demonstrated that the complex additives in CXI cements significantly supported the maturation and osteogenic activity of osteoblasts cultured in 50% cement extracts. 

## Figures and Tables

**Table 1:** Composition of cement samples.

Sample	P/L Ratio	PAA Content/wt%
CXI1	1.7	0.5
CXI2	2	0.5
CXI3	1.7	1
CXI4	2	1
CX	2	free of PAA, CMC
C *	2	free of PAA, CMC, PHYT

* 2% NaH_2_PO_4_ hardening liquid.

**Table 2:** Forward and reverse primers of genes used for RT-qPCR experiments.

Gene	Primers (5′–3′)	Length (bp)	Reference
**B-actin mouse**	F: CTCTGGCTCCTAGCACCATGAAGA R:GTAAAACGCAGCTCAGTAACAGTCCG	200	[35]
**Osteocalcin** **mouse**	F: AGCAGGAGGGCAATAAGGTAGT R: TCGTCACAAGCAGGGTTAAGC	118	[36]
**Osteopontin** **mouse**	F: TGATTCTGGCAGCTCAGAGGA R: CATTCTGTGGCGCAAGGAGATT	110	[36]
